# How to customize a bona fide psychotherapy for generalized anxiety disorder? A two-arms, patient blinded, ABAB crossed-therapist randomized clinical implementation trial design [IMPLEMENT 2.0]

**DOI:** 10.1186/s12888-018-1666-2

**Published:** 2018-04-03

**Authors:** Christoph Flückiger, Christine Wolfer, Judith Held, Peter Hilpert, Julian Rubel, Mathias Allemand, Richard E. Zinbarg, Andreea Vîslă

**Affiliations:** 1 University of Zürich, Zürich, Switzerland; 2 University of Trier and Northwestern University, Evanston, USA; 3 University of Zürich and Trier, Zürich, Switzerland; 4 Department of Psychology, University of Zürich, Binzmühlestr. 14/04, -8050 Zürich, CH Switzerland

**Keywords:** Generalized anxiety disorder, Cognitive behavior therapy, Sudden gains, Early change, Responsiveness, Therapist effects, Personalized medicine, Randomized clinical trial, Evidence-based practice, Translational science

## Abstract

**Background:**

Bona fide psychotherapy approaches are effective treatments for generalized anxiety disorder (GAD) compared to no-treatment conditions. Treatment manuals and protocols allow a relatively high degree of freedom for the way therapists implement these overall treatment packages and there is a systematic lack of knowledge on how therapists should customize these treatments. The present study experimentally examines two implementation strategies of customizing a bona fide psychotherapy approach based on a 16 session time-limited cognitive-behavioral therapy (CBT) protocol and their relation to the post-session and ultimate treatment outcomes.

**Methods:**

This trial contrasts two different implementation strategies of how to customize the in-session structure of a manual-based CBT-protocol for GAD. The patients will be randomly assigned to two implementation conditions: (1) a systematic focus on subtle changes lasting from 7 to 20 min at the check-in phase of every psychotherapy session and (2) a state-of-the-art (SOTA) check-in phase lasting several minutes mainly focused on the session goals. Potential therapist effects will be examined based on an ABAB crossed-therapist design. Treatment outcomes will be assessed at the following times: post-session outcomes, treatment outcome at post assessment and 6- as well as 12-month follow-up.

**Discussion:**

The proposed randomized clinical implementation trial addresses the clinically relevant question of how to customize a bona fide psychotherapy protocol experimentally contrasting two implementation strategies. Through the development and testing of the proposed implementation design, this trial has the potential to inform therapists about efficacious implementation strategies of how to customize a manual-based treatment protocol in respect to the timing of the in-session structure.

**Trial registration:**

This trial was registered at ClinicalTrials.gov (NCT03079336) at March 14, 2017.

## Background

Personalized medicine refers to several efforts of tailoring interventions to the characteristics of each individual patient (e.g. [1, 2]). For psychotherapy, one can argue, that personalization of treatments is already part of the clinical reality of most practitioners (e.g. [3, 4]). However, evidence-based guidelines of how to customize treatments and how to more specifically structure the sessions are largely missing. This might in part be due to the fact that in psychotherapy and in human treatments more generally, not only does patient heterogeneity need to be taken into account, but potentially also that of the therapist and their collaborative qualities between each other.

Within the over 25,000 hits in relevant data bases during the past 5 years referring to *randomized controlled trial* design in *human interventions*, based on a search at February 2018 in Medline, PsychINFO and ERIC, there is a lack of sensitivity to heterogeneity in therapists’ effectiveness (i.e. therapist effects) and a lack of systematized knowledge about how therapists implement their treatments [5]. There is a need for further work developing and testing study designs that investigate therapist effectiveness and related therapists’ implementation strategies [6–9].

The present study design represents an important step toward addressing this gap by investigating two implementation strategies along therapist and patient variability with a bona fide psychotherapy approach for individuals that suffer from generalized anxiety disorder (GAD). We are conducting a novel two-arms, patient blinded, ABAB crossed-therapist randomized controlled implementation trial design.

### Therapist effects in randomized clinical trials

Blinded allocation of patients and health professionals is an important claim in double-blind randomized pharmaceutical trials. It is a well-known consideration for the identification of a true treatment effect and for avoiding potential confounding effects from patients and health professionals. In evidence-based human interventions, health professionals can not be blinded, because hopefully they are well-informed and fully aware of the interventions that they apply (e.g. the professionals apply a specified surgery, educational program, or psychological intervention). Such conditions are common in human interventions of evidence-based medicine [10], nursing [11], social work [12], education [13], and psychological interventions more generally [5, 14].

There are at least three positions regarding the debate about non-blinded therapist conditions and how to handle potential therapist effects in human interventions: (1) advocates for double-blind trial designs highlight *general biases* of non-blinded allocations, assuming that blinded conditions may help to eliminate potential confounding effects (e.g. [15]), (2) advocates of evidence-based human interventions often emphasize *uniformity of evidence-based treatments* without considering therapist effects as a potential confounder, i. e. potential therapist effects often are neglect at the analyses and discussion sections when conducting study designs where patients are nested in therapists (see [16]), and (3) a third way to investigate therapist effects either in non-blinded as well as blinded conditions lies in the direct investigation of these potential effects and the development of study designs that allow for the estimation of such effects; i.e. therapist effects considered as *true effects* rather than biases or confounders (see [5, 17]).

Crossed-therapist designs were proposed for naturalistic intervention studies [18, 19], where each therapist is allocated into two or more treatment conditions, and therefore potential differences in overall therapist effectiveness can be estimated across conditions. However, the therapists’ individual treatment preferences for a certain condition may impact her effectiveness across conditions, and thus, therapist preferences should be explicitly assessed in such designs [5, 19].

### Generalized anxiety disorder

Randomized controlled trials usually are focused on one particular patient population (e.g. by specifying disorders or particular contexts of suffering) to reduce the patient variability and to enhance the precision of research results. Uncontrollable worry is a primary symptom of GAD and constitutes a maladaptive cognitive strategy to avoid the experience of anxiety [20] and other emotional states [21, 22]. Individuals who suffer from GAD show deficits in detecting and regulating emotional states, which might accelerate a positive feedback circuit between general stress symptoms and pathological worrying (e.g. [23]). Experiential avoidance might lead to a restriction in proactive behaviors because individuals become focused on preventing negative events and maintaining safety [24], rather than on pursuing activities that are consistent with their personal values (e.g. [25]), which might impact the content of psychotherapy sessions [26].

### Empirical evidence of bona fide psychotherapy approaches for GAD

There is meta-analytic evidence that psychotherapy conditions conducted by trained professionals that are designed to be fully therapeutic (bona fide psychotherapy; [5, 27]) are more effective treatments compared to no-treatment and treatment as usual for individuals with GAD [28–30] as well as for individuals who suffer from anxiety and depression comorbidities more generally [27, 31]. For cognitive-behavioral therapy (CBT), there are a number of GAD-specific interventions, such as GAD-psychoeducation, applied relaxation, restructuring of (meta-) cognitions, (imagery-) exposure, and in vivo confrontation, that primarily reference standard techniques to reduce and compensate GAD symptoms (e.g. [32–39]; but also [40]). In daily practice, however, it is a well-known fact that patients and therapists usually are not uniformly skilled. On a broader perspective psychotherapy dialogues were observed to be highly collaborative and responsive treatments, through which *therapists as well as patients* work together to achieve well-specified treatment goals that consider the patients’ entire living environment [41–44].

### Therapist’s responsiveness to subtle patient changes

It is a robust finding in psychotherapeutic as well as pharmacological mental health treatments that sudden patient’ changes commonly occur independently of the intake severity. For example, sudden symptom changes (sudden gains) occur in approximately 20–40% of all clients, up to 60–80% in those who benefit from treatment in general [45–49], and in GAD more specifically [50, 51]. Whereas such research focuses on sudden symptom reduction of particular symptoms [52, 53], other investigators highlight psychological changes involving other aspects of a comprehensive definition of health [54] including substantial as well as more subtle changes in wellbeing and psychosocial functioning [47, 55–59].

From a therapist’s implementation perspective, detecting and integrating subtle patient changes, even small and subtle ones, in a more systematic manner might impact the treatment processes and its outcomes [46, 59]. It is likely that many therapists already respond to sudden gains and subtle changes, and in this way, help to regulate the “speed” of therapeutic change and the consolidation of gains achieved [47, 60]. Even though subtle changes seem to occur in many psychotherapies, to the best of our knowledge, there is no trial that systematically takes advantage of such changes using rigorous randomized controlled trial methodology. There is a need for future research in this area, especially because subtle changes are apparent in many mental health conditions [45–47, 57, 59, 61–63].

In a prior three-arm, single-blinded, randomized controlled implementation trial (IMPLEMENT 1.0; [64]) we recruited 57 adults with GAD to participate in a cognitive behavioral treatment approach very similar to the approach used in our current trial. We randomly assigned eligible patients to three different priming conditions: (1) adherence priming, in which the peer-priming with the therapist had a systematized focus on patients’ individual GAD symptoms and how to compensate for these symptoms within the manual, (2) resource priming, in which the peer-priming with the therapist had systematized foci on patients’ strengths and abilities and how these strengths can be capitalized within the same package and (3) supportive resource priming (that additionally allowed the invitation of a supportive person into therapy sessions). The results indicated that all three priming conditions showed a highly significant reduction of symptoms over time. However, compared with the adherence priming condition, both resource priming conditions indicated faster symptom reduction during treatment. In contrast to this past trial where the implementation conditions focused on different peer-priming strategies, in the present implementation trial we focus on contrasting two implementation strategies that focus on taking different approaches to subtle change made by the patient.

### Aims of the implementation trial

Rather than contrasting increasing numbers of new overall treatment-packages, an additional approach may be to increase the understanding of fine-grained implementation strategies within already effective psychotherapies. Such research questions require the development of novel randomized clinical implementation designs that simultaneously investigate potential implementation effects along with therapist as well as patient effects [5].

This trial investigates two different implementation strategies of customizing a bona fide psychotherapy based on a well-introduced CBT-protocol for GAD [39]. The patients will be randomly assigned to two implementation conditions: (1) a systematic focus on subtle changes lasting from 7 to 20 min at the check-in phase of every psychotherapy session [61, 65]; and (2) a state-of-the-art (SOTA) check-in phase lasting several minutes mainly focused on the session goals. Potential therapists’ implementation effects will be examined based on an ABAB therapist allocation (see design). The main research questions are as follows:

1. Practicability: Is the newly developed randomized clinical implementation trial design and particularly the therapists’ ABAB allocation practicable (ABAB crossed-therapist design), and what are the specific challenges when conducting such a structured design at the patient as well as therapist level?
2. Post-session outcomes: Are there differences in process evaluations measured by post-session reports for the two implementation conditions? Furthermore, are early post-session outcomes predictors of symptom change and mediators of ultimate therapy outcome (e.g. [66])?
3. Treatment outcomes: What are the differences in the treatment efficacy of the two randomized implementation conditions, (1) in dropout rates, (2) in self-reported primary and secondary outcomes [64]?
4. Therapist effects: Are the implementation effects robust across therapists? Is there an interaction effect between potential therapists’ preferences for a particular implementation condition and outcome?

## Methods/design

### Design

**Fig. 1:**
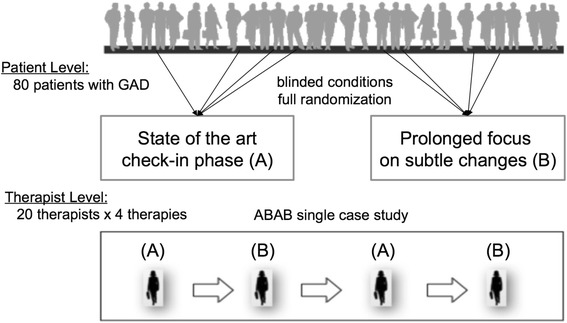
Randomized, clinical implementation trial design: Patient and therapist allocation

It is a common data structure in human interventions that patients are nested within therapists. The proposed randomized controlled design systematize both levels, i. e. patient as well as therapist level (Fig. 1 for the nested design):

1. At patient level: Eighty patients will be allocated within a traditional 2 × 4 randomized controlled design with one between-subject factor (systematic focus on subtle changes vs. SOTA check-in phase) and one within-subject factor (assessment times: pre-, intermediates-, post-treatment and follow-ups).
2. At therapist level: Twenty therapists will each conduct four therapies using an ABAB-design randomly starting with a SOTA check-in phase or a systematic focus on subtle changes (80 patients). Overall, this ABAB crossed-therapist design allows us to integrate across what might be considered single case studies of 20 therapists [67]. An advantage of this design is, that within-subject effects can be estimated. However, multiple observations may also lead to learning effects, and potential carry over effects may be detected from the therapist’s first to the fourth therapy. Most importantly, however, such therapist effects are not considered as effects that have to be eliminated. Rather, the longitudinal, multilevel design allows us to estimate therapist effects in parallel to the implementation effects [5].

### Participants

1. Inclusion/exclusion criteria: Participants will be included in the study if they: (1) are 18 years or older; (2) agree to the informed consent, (3) can speak German; and (4) fulfill the diagnostic criteria of GAD DSM-5. Participants will be excluded for the following reasons: (1) they have a score of 2 or higher on the suicide item of the Beck Depression Inventory and/or are found to have active suicidal plans during the diagnostic screening interview, (2) they are currently taking a psychotic or bipolar disorder medication, or (3) they are currently receiving treatment from a professional psychotherapist. Prescribed medications for anxiety or depressive disorders do not lead to exclusion from the study, if the dosage has remained constant for at least 1 month. The presence of comorbidities does not result in exclusion from the study, if GAD is in the foreground according to the severity rating of the Diagnostic Interview for DSM-diagnoses.
2. Recruitment: Participants will be recruited by e-mail distributors of internet forums. Individuals interested in participating in the study will contact the study office via SMS, e-mail or phone. Positively screened patients will be invited for an intake assessment to determine whether they will be included or excluded using a standardized diagnostic interview. Participants that are not screened positively will be informed of more appropriate treatments via a phone call or, if requested, a face-to-face contact.
3. Randomization and treatment allocation. After meeting the inclusion criteria, patients will be randomly assigned to one of the two implementation conditions (systematic focus on subtle changes or SOTA check-in phase). Treatment allocation is performed using an online application for full randomization. The whole randomization procedure will be conducted by an independent person outside of the project implementation. In this way, we aim to ensure that the trial arms are fully randomized with respect to the patients’ baseline characteristics. Because all patients will be treated using the same CBT-manual, patients are *blinded* to their treatment allocation and are not informed about the randomization procedure.

### GAD treatment protocol

Modern CBT for GAD typically consists of psychoeducation, relaxation training and/or mindfulness exercises (RT/M), cognitive restructuring (CR) and imagery exposure (IE) as major interventions to address the various GAD-related problems. The “Mastery of your Anxiety and Worry” manual (MAW-package) is a well-structured, and internationally well-introduced cognitive-behavioral treatment [39, 68]. In the present study, the MAW-package will be applied within a usual 16-session individual therapy format and up to three further booster sessions. The treatment protocol was based on a 16-h workshop presented by one of the co-developers of the treatment manual. The MAW-package consists of (1) psychoeducation regarding the nature of GAD and the rationale for the treatment program (sessions 1 and 2), (2) RT/M (sessions 3 and 4 and portions of sessions thereafter), (3) CR (sessions 5, 6 and 7 and portions of sessions thereafter), (4) IE (sessions 8–12), and (5) plans for maintenance, relapse prevention and termination (sessions 13–16). However, the implementation of an bona fide treatment is largely principle-based, allowing considerable therapeutic flexibility in determining and timing of different treatment aspects.

The session format of 50–60 min usually consists of (1) a check-in phase of 5 to 10 min that includes patients welcoming, reviewing self-help and agenda setting, (2) a working phase around 35–45 min that focuses on the previously agreed session goals, (3) a feedback phase of 5 to 10 min that summarizes the session and includes a negotiation of the upcoming self-help assignment.

### Implementation conditions: In-session structure

The session check-in phase will be systematically varied by the following two conditions while keeping the overall session lengths of 50–60 min constant:

1. State-of-the-art (SOTA) check-in phase: The therapists will apply the usual SOTA check-in phase lasting between 5 to 10 min, as recommended in the preexisting guideline including reviewing progress in self-help and agenda setting [39]. In this condition progress, subtle changes and sudden gains may be an explicit topic and there is no restraint to talks about topics. However, the therapists are not obligated to take a systematic focus on potential subtle changes and they may use the timing of the sessions to involve the patients into the other phases of therapy.
2. Prolonged focus on subtle changes: Based on the robust findings that over 90% of the patients will experience subtle changes [69, 70] e.g. apparent at the pre-session assessments, the therapists will extend the above-mentioned check-in phase to 7 up to 20 min in which small and subtle changes are systematically worked with. These subtle changes will further be explored by focusing on the following aspects [61, 71]: (1) the precise change situation, (2) related emotional states, (3) related helpful thoughts and self-verbalizations, (4) reinforcement of generalized self-efficacy and treatment motivation, (5) benefit for the upcoming session goals. Exploratory video analyses of GAD patients indicated that the explicit examination of the patients’ changes and immediate competencies usually observed at the check-in phase was highly associated with therapy success [26].

### Therapists

Twenty psychologists with a master degree and participating in post-graduate psychotherapy trainings are recruited from local psychotherapy-training centers. In addition to the clinical internships, these systematized 5-year postgraduate training includes (1) 600 h of weekend workshops for psychotherapy interventions in single, couple, and group settings, (2) 200 h of supervision, and (3) 200 h of personal therapy. Some of those therapists have experience as study administrators in a prior randomized controlled trial for GAD (ClinicalTrials.gov Identifier: NCT02039193). All therapists will participate in an initial 16-h workshop presented by one of the co-developers of the treatment manual (Zinbarg) [39]. The therapists will be regularly supervised in small groups on a bi-weekly basis. The supervision is conducted in mixed groups for all conditions. All supervisors also participate in the initial 16-h workshop. For a therapist performing an ABAB sequence of conditions, the therapy will be performed as followed: First therapy in the SOTA-check-in phase condition, second therapy in the prolonged focus on subtle changes condition, third therapy with the SOTA check-in phase condition, forth therapy with the prolonged focus on subtle changes condition (ABAB sequence); the other half of the therapists will start with the prolonged focus on subtle changes condition (BABA sequence).

### Assessments

For an overview of the assessments see Table 1. At intake, GAD-diagnosis and its core symptomatology is identified according to the structured interview section for GAD (DIPS; [72]). Furthermore, GAD-criteria are assessed using self-reports. The individual worries are identified using the Penn State Worry Questionnaire (PSWQ; [73]) and the Worry Domain Questionnaire (WDQ; [74, 75]). Mental disorders on Axis I are assessed using face-to-face diagnostic interviews (Strukturiertes Klinisches Interview für DSM, SKID-I; [76]).

1. GAD-specific (primary) outcomes: The Penn State Worry Questionnaire (PSWQ; [73]) is a 16-item measure of the frequency and intensity of worry. It has considerable internal consistency (α = 0.86 in the prior trial). The Beck Anxiety Inventory (BAI; [77]) is a 21-item measure for anxiety symptoms (α = 0.92 in the prior trial).
2. General (secondary) outcomes: The Beck Depression Inventory II (BDI-II; [78]) is a 21-item measure for depressive symptoms (α = 0.92 in the prior trial). The 9-item short version of the Symptom Check List (SCL-9; [79]) is an index for general severity (α = 0.86 in the prior trial). The short version of the Resource Self-Report Questionnaire (RES; [80]) measures various domains of individual and interpersonal strengths (α = 0.92 in the present sample).

**Table 1:** Assessments

Measures	Assessment time					
	Recr.	Pre	S-by-S	Int.	Post	FU
Eligibility						
Structured Interview for DSM (SCID)	+					
GAD-diagnosis (DIPS)	+					
Worry Domains Questionnaire (WDQ)	+					
GAD-outcomes						
Beck Anxiety Inventory (BAI)		+		+	+	+
Penn State Worry Questionnaire (PSWQ)	+	+	1–16	+	+	+
General-outcomes						
Premature termination			1–16	+	+	+
Beck Depression Inventory (BDI)	+	+		+	+	+
Brief Symptom Inventory (BSI)		+		+	+	+
Resource potential questionnaire (RES)		+		+	+	+
Self-report process-measures						
Working Alliance Inventory - Patient (WAI-P)			1–16			
Working Alliance Inventory – Therapist (WAI-T)			1–16			
Bern Post-Session Report – Patient (BPSR-P)			1–16			
Bern Post-Session Report – Therapist (BPSR-T)			1–16			
Patients’ Therapy Expectation and Evaluation (PATHEV)		+		+		
Therapists’ preferences and outcome expectations	+ _1_					
Therapists’ self-reported focus on subtle changes			1–16			

_1_At the beginning of the study, _2_ At the end of the study, *Recr*. Recruitment, *Pre* Intake assessment, *S-by-S* Session by session assessment, *Int.* Intermediate assessment (at session 5 and 10), *Post* Post assessment, *FU* Follow-up assessments (at 6- and 12-months after treatment termination)

In the prior trial, all GAD and general outcome self-report assessments were highly correlated (0.74 > *r* > 0.47) and a principal component factor analysis extracted one component that explained 67.5% of the total variance. For the purposes of the present trial, we therefore intend to include a standardized composite measure (“outcome composite”) that takes all the five GAD and general self-report measures into account (α = 0.73 in the prior trial; see also [81] for psychotherapy outcome definitions).

(3) Post-session outcomes: The following process measures are examined: (a) Post-session outcomes (evaluated by therapists and patients): Working Alliance Inventory (WAI) [82, 83], Bern Post-Session Reports (BPSR) [84], and PSWQ patient self-report symptom status are conducted based on a session-by-session assessment from session 1 to 16. Furthermore, patient and therapist outcome expectations as well as therapist implementation preferences and in-session focus on subtle change will be assessed based on self-report items.
(4) Safety outcomes: Suicidal ideation is assessed as safety outcome in the structured assessment at baseline, post, and follow-up measures and if indicated in every therapy session. As an outcome, suicidal ideation is assumed when the suicidal ideation item of the BDI is endorsed with a value > 1. In this case, individuals will be immediately contacted by telephone for further assessment and coordination as well as decisions for more appropriate treatments will be based on the general psychotherapeutic guidelines for suicidal ideation [85].

### Statistical analysis

Along more traditional statistical approaches to handle the nested data structures, Longitudinal Multilevel Modeling (MLM) will be used to analyze the (intensive) longitudinal, nested data structures [86–89]. This data-analytical approach is specifically suitable to analyze the type of data that will be collected in the proposed implementation trial. For the primary research questions for post-session and treatment outcomes, MLM with time as a repeated within-group factor and implementation condition as a between-groups factor will be used [90]. A benefit of MLM, in comparison to traditional ANOVA with repeated measures lies in the individual parameterization of the process variables over various levels (repeated measures as random variables; [89]). The main analyses will be conducted on the intention-to-treat sample and the completer data. Between-group effect sizes will be based on pre-post within control Cohen’s *d*_ppwc_ suggested by Carlson and Schmidt [91] which is a mean difference estimate that takes into account both intake values at pretreatment and differences between each treatment condition.

#### Statistical exemplification

The following analysis estimates the treatment efficacy of the two implementation conditions using random effects multilevel models [89]. To consider the variance components at each level (assessment times nested in patients and therapists), we will conduct 3-level models where the repeated assessments of the primary outcome at level 1 will be nested within patients at level 2 and therapists at level 3 (for a comparable model see e.g. [92]). Using a step-by-step approach [91], the growth models potentially will end up in a multi-predictor model to investigate the main outcome-predictors at each level simultaneously. An example of such a model is: At Level 1 (assessment level),

*PSWQ*_*ijk*_ = *π*_0*jk*_ + *π*_1*jk*_(time) + *e*_*ijk*_

where *PSWQ*_*ijk*_ is the estimated value of the Penn State Worry Questionnaire (PSWQ) within a time-specific assessment of each patient_j_ and therapist_k_; *π*_*0jk*_ represents the intake-centered intercept of the repeated PSWQ-measures within each patient; *π*_*1jk*_ (time) represents the decrease of symptoms from pretreatment up to post-treatment, and *e*_*ij*_ represents the corresponding error term.

At Level 2 (patient level),

for intercept: *π*_0*jk*_ = *ß*_00*k*_ + *ß*_01*k*_(number of comorbidities) + *r*_0*jk*_.

for time: *π*_1*jk*_ = *ß*_10*k*_ + *ß*_11*k*_(implementation condition) + *r*_1*jk*_*.*

where *π*_*0jk*_ is the estimate of the true population overall patients’ intake PSWQ and *π*_*1jk*_ is the estimation of the overall symptom reduction across the repeated assessments. Further, *ß*_*00k*_ and *ß*_*10k*_ are the estimated overall grand means of PSWQ intercept and time, and *ß*_*01k*_(number of comorbidities) and *ß*_*11k*_(implementation condition) represent the specific patients’ level predictors on intercept *π*_*0jk*_ and growth *π*_*1jk*_ respectively*.* Finally*, r*_*0j*k_ and *r*_*1jk*_ are the corresponding error terms at the patients’ level.

At Level 3 (therapist level):

*ß*_00*k*_ = *γ*_000_ + *u*_00*k*_

*ß*_01*k*_ = *γ*_010_

*ß*_10*k*_ = *γ*_100_

*ß*_11*k*_ = *γ*_110_ + *γ*_111_(therapists^’^ implementation preference) + *u*_11*k*_

where *ß*_*00k*_ is the estimate of the true population therapists’ overall intake PSWQ and *ß*_*10k*_ is the corresponding overall true population symptom reduction *among therapists*; *ß*_*01k*_ and *ß*_*11k*_ are the overall therapists’ true population estimations of the level 2 predictors. Furthermore, *γ*_*000*_ and *γ*_*100*_ are the estimated overall grand means of PSWQ intercept and time at therapists’ level, *γ*_*111*_ (therapists’ implementation preference) is the hypothesized therapists’ preference on the implementation condition *ß*_*11k*_. Finally, *u*_*00k*_ and *u*_*11k*_ are the corresponding error terms at the therapist level (see also [6, 93] for a conceptual frame).

The various multi-predictor models will be compared using Akaike information criterion (AIC) and the Bayesian information criterion (BIC; see [88]) and deviance test of variance components respectively [89].

### Power considerations

With respect to the differences between the two comparative conditions, based on prior studies with transdiagnostic disorders [94], social phobia [95], depression [96], and GAD [64], we planned to demonstrate a medium effect size of Cohen’s *d* = 0.40 between the implementation conditions on the outcome composite. Assuming an α error level of 0.05, a statistical power (1-Beta) of 0.80, and a correlation of 0.40 between the pre- and post-measurements, the proposed study of 40 participants in each condition treated by 20 therapists is able to detect such an effect size [97].

## Discussion

Treatment protocols allow a relatively high degree of freedom for the way therapists implement the treatment protocols. The present design is one of the very first trials that experimentally examine the therapists’ sensitivity to changes and its relation to treatment outcome. More specifically, two implementation strategies of session structuring (SOTA check-in phase, prolonged focus on subtle changes) are compared to each other in the same overall treatment package.

The hierarchical structure of the systematized design allows the simultaneous examination of patients’ and therapists’ contributions. In contrast to pharmaceutical trials, therapists and patients are informed and (hopefully) proactively involved in the psychotherapeutic treatment. This involvement is not a bias which has to be eliminated; it might rather be an active ingredient of a successful psychotherapy in which the therapists and the patients take a proactive and collaborative role in the treatment plan [41]. Therefore, the present design allows to experimentally investigate some potentially meaningful aspects of this responsive proactivity.

### Bias minimization

The hierarchical structure of the implementation design allows a systematic investigation of patients’ as well as the therapists’ contributions simultaneously; and in one further implementation aspect, it allows to examine an experimental contrast (SOTA check-in phase vs. prolonged focus on subtle changes check-in phase). But for the similarities and differences of the proposed randomized clinical implementation trial with the common randomized clinical trial design see Table 2.

1. Patients*:* Patients will be randomly assigned to conditions to reduce systematic selection biases in participant characteristics. The inclusion and exclusion criteria allow for a relatively homogeneous group of individuals with GAD diagnoses. In traditional trials, where two or more distinctive treatment protocols are compared to each other, patients have to be informed about the various randomized treatment conditions of the active treatments (e.g. psychotherapy vs. waiting list). Therapist will apply the very same bona fide psychotherapy protocol in both conditions. Implementation-strategies are part of the therapist individual case preparation. Based on common practice, therapists are not obligated to inform the patient about any specific topic of therapist case preparation, related supervision, or personalized implementation strategies. Therefore, implementation condition can be blinded for the patient at every time of the study conduction. This is one of the very first designs that allows for blinded patient conditions which minimizes some of the potentially major concerns of the more traditional randomized controlled trials of psychotherapy and psychological interventions more generally.
2. Therapists*:* A potential bias due to the therapist preferences is a concern, especially in human treatments where inductions of outcome expectations can be impactful for patients as well as therapists. In the proposed design, potential therapist effects are not considered as a bias that has to be eliminated. In contrast, due to the nested data structure, the present ABAB crossed-therapist design is able to take into account such possible effects [6]. Furthermore, this design allows systematizing potential implementation effects *within* therapists.
3. Researchers*:* The existence of researcher allegiance effects is a robust meta-analytic finding in both comparative (e.g. [98]) and correlative designs (e.g. [99]). In many randomized controlled trials, maximally distinctive treatment protocols are contrasted with each other (e.g. cognitive behavioral therapy vs. psychodynamic therapies; online vs. face-to-face treatments, additive components vs. standard treatment). Imbalances in researcher allegiance might favor one treatment, sometimes very explicitly and sometimes more subtly. For example, some patients might have been attracted by a specific intervention and therefore self-selected the specialized clinic, but were randomized in the alternative treatment. Another example is that the designed control groups are intended to not be fully therapeutic [not bona fide, 5]. Even though we presented some literature of how to innovate state-of-the-art CBT (what might be an indicator for researcher allegiance in favor to the prolonged focus on subtle changes condition), both interventions are designed to be fully therapeutic within the very same bona fide treatment package. Therefore, the aim of the present investigation is not to contrast distinctive treatments packages. The aim is rather to keep the overall treatment package constant in order to investigate a relevant clinical research question of treatment implementation (i. e. therapist consideration of subtle patient change) *within* a treatment package. Nonetheless**,** despite the awareness of potential researcher allegiance effects in our research team, it might be difficult to fully eliminate such effects over the course of the lasting study implementation. Hence, during the publication process we will discuss this potential limitation cautiously.

**Table 2:** Similarities and differences of randomized controlled trial and randomized clinical implementation trial designs in human interventions

Design:	Randomized clinical trial	Randomized clinical implementation trial
Treatment manual / protocol:	Contrast between different packages (e.g. comparative, additive, subtractive designs)	Same package over all conditions, contrasts between implementation strategies (e.g. timing, sequence, focus)
Patient allocation:	Randomized, not blinded	Randomized, blinded
Therapist allocation:	Not randomized, not blinded	Randomized or systematic allocation (e.g. ABAB), not blinded
Researcher allegiance:	Substantial	Not investigated yet

To conclude, an essential contribution of this study will be to better understand successful implementation strategies of how to customize a manual-based psychotherapy in respect to the session timing. In addition, the present randomized controlled implementation trial may provides further insights about therapist effects based on a ABAB crossed-therapist allocation where patients can be fully blinded about their implementation condition. Most relevant, the present study protocol may sensitize for potential implementation effects when conducting randomized controlled trials in human interventions.

## Abbreviations

BAI: Beck anxiety inventory
BDI-II: Beck depression inventory - version II
BPSR: Bern post-session reports
CBT: Cognitive behavioral therapy
CR: Cognitive restructuring
DSM-5: Diagnostic and statistical manual of mental disorders - version 5
GAD: Generalized anxiety disorder
IE: Imagery exposure
MAW-package: Mastery of your anxiety and worry package
PSWQ: Penn state worry questionnaire
RES: Resource self-report questionnaire
RT/M: Relaxation training and/or mindfulness exercise
SCID: Structured clinical interview for dsm-diagnostics
SCL: Symptom check list
SOTA: State-of-the-art
WAI: Working alliance inventory
WDQ: Worry domain questionnaire
